# Morning Versus Evening Exercise for Blood Pressure Control in Hypertension: A Systematic Review of Randomized Controlled Trials

**DOI:** 10.7759/cureus.107068

**Published:** 2026-04-14

**Authors:** Shekha Alnajdi, Abdullah Alkandari, Abdillatef Alqemlas, Aesha Ali Alasade, Abdulrahman M Aldousari, Salah J AlRashidi, Areej M Basheer

**Affiliations:** 1 Internal Medicine, Faculty of Medicine, The University of Jordan, Amman, JOR; 2 Internal Medicine, College of Medicine, Farwaniya Hospital/Ministry of Health, Farwaniya, KWT; 3 Internal Medicine, Al-Sabah Hospital, Kuwait City, KWT; 4 Internal Medicine, Jahra Hospital/Ministry of Health, Al Jahra, KWT

**Keywords:** blood pressure, exercise, hypertension, randomized clinical trials, systematic review

## Abstract

Hypertension remains the leading modifiable risk factor for cardiovascular morbidity and mortality worldwide. While clinical guidelines strongly recommend lifestyle modifications, including exercise, as a core treatment, they currently lack specific recommendations regarding the optimal timing of its administration. Given the circadian regulation of blood pressure (BP), autonomic tone, and vascular function, the timing of exercise may impact BP control. This systematic review aims to synthesize evidence from randomized clinical trials (RCTs) to compare the effects of morning and evening exercise on BP control in hypertensive patients. A systematic literature search was conducted using PubMed, Scopus, Web of Science, and Embase from inception to January 2026. We included RCTs involving adult hypertensive patients that compared morning and evening exercise interventions. Outcomes of interest included systolic BP (SBP), diastolic BP (DBP), mean arterial pressure, 24-hour ambulatory BP, nocturnal BP, and post-exercise hypotension (PEH). Five studies, comprising four trials (two long-term parallel RCTs and two short-term crossover RCTs), were included. Long-term interventions indicated that evening exercise was superior to morning exercise in reducing SBP and mean arterial pressure (MAP) in hypertensive men. Short-term interventions yielded conflicting results. However, limited data suggest that morning exercise may be more beneficial for women in reducing SBP and attenuating stress reactivity. The timing of exercise may influence BP control in hypertensive patients. Evening exercise appears to be the optimal strategy for long-term BP reduction in hypertensive men. Conversely, morning exercise may be preferable for women. These findings suggest that exercise recommendations should be tailored individually to each patient. Future efforts should be directed towards long-term studies to better elucidate the effects of morning and evening exercise on BP control.

## Introduction and background

Hypertension remains the single most significant modifiable risk factor for cardiovascular disease and premature mortality globally. According to the World Health Organization (WHO), an estimated 1.4 billion adults aged 30-79 years worldwide are currently affected by hypertension, accounting for approximately 33% of the global population within this age group [1]. Despite the widespread availability of pharmacological treatments, blood pressure (BP) control remains suboptimal; nearly four out of five hypertensive individuals do not achieve recommended targets, leaving them vulnerable to stroke, myocardial infarction, and renal failure [2]. Current clinical guidelines, including the 2024 recommendations from the European Society of Cardiology (ESC) and the American Heart Association (AHA), strongly recommend lifestyle modifications, including structured aerobic and isometric exercise, as an essential part of treatment [3,4]. However, while these guidelines provide details regarding the frequency, intensity, and duration of exercise, they offer no guidance regarding the optimal timing of its administration. 

The cardiovascular system is regulated by a circadian rhythm, an internal biological clock that regulates fluctuations in heart rate, vascular tone, and BP over 24 hours [5]. In healthy individuals, BP exhibits a distinctive "dipping" pattern, declining by 10%-20% during nocturnal sleep to reduce hemodynamic stress on the heart and kidneys [6]. In contrast, the transition from sleep to awake is marked by a morning surge; a fast rise in BP driven by sympathetic activation and a spike in cortisol and catecholamines [7]. Disruptions in this rhythm are potent predictors of adverse outcomes. The "non-dipping" phenotype is strongly associated with target organ damage and increased cardiovascular mortality. Similarly, an exaggerated morning surge increases the risk of plaque rupture and ischemic events during the early hours of the day [8]. Therefore, interventions that can specifically target these high-risk windows, restore nocturnal dipping, or blunt the morning surge are of paramount clinical importance. 

The concept of chronotherapy states that the timing of a therapeutic intervention can significantly influence its efficacy. Regarding the exercises, post-exercise hypotension (PEH), the sustained reduction in BP following a single bout of exercise, is a well-documented phenomenon that contributes to chronic BP control [9]. However, emerging evidence suggests that the magnitude and duration of PEH may be time-dependent. Biological variances in vascular resistance, autonomic tone, and endothelial function throughout the day may create specific "windows of opportunity" where exercise is most effective [10]. For instance, evening exercise may coincide with the natural circadian decline in sympathetic tone, potentially amplifying vasodilation and extending hypotensive benefits into the sleep period [11].

Despite the theoretical promise of exercise chronotherapy, the existing literature presents conflicting findings. The relationship between exercise timing and patient characteristics, such as age, medication use, and dipping status, remains underexplored in broad clinical guidelines. This systematic review aims to synthesize the available evidence of randomized clinical trials (RCTs) regarding the differential effects of morning versus evening exercise on BP in hypertensive patients.

## Review

Methods

Literature Search

This systematic review was conducted and reported in accordance with the PRISMA 2020 guidelines. A comprehensive literature search was performed using PubMed, Scopus, Web of Science, and Embase from database inception to January 2026. The search strategy included terms related to hypertension, exercise, and timing of exercise. The full search strategy for each database is provided in Table 1. Additionally, reference lists of included studies were manually screened to identify any additional relevant studies. 

**Table 1:** Detailed search strategy for each database during the systematic search phase.

Database	Search Strategy	Filter	Results
PubMed	(“Hypertension” OR “Hypertensive” OR “HTN” OR “Elevated blood pressure” OR “High blood pressure” OR “Hypertensia” OR “Arterial hypertension” or “Systemic hypertension”) AND (“Morning” OR “Daytime” OR “Sunlight” ) AND (“Evening” OR “Midnight” OR “Darkness” OR “Nighttime”) AND (“Exercis*” OR “Train*”)	All fields	N = 201
Scopus	TITLE-ABS-KEY("Hypertension" OR "Hypertensive" OR "HTN" OR "Elevated blood pressure" OR "High blood pressure" OR "Hypertensia" OR "Arterial hypertension" OR "Systemic hypertension") AND TITLE-ABS-KEY("Morning" OR "Daytime" OR "Sunlight") AND TITLE-ABS-KEY("Evening" OR "Midnight" OR "Darkness" OR "Nighttime") AND TITLE-ABS-KEY(Exercis* OR Train*)	Article title, abstract, keywords	N = 220
Embase	('Hypertension':ti,ab OR 'Hypertensive':ti,ab OR 'HTN':ti,ab OR 'Elevated blood pressure':ti,ab OR 'High blood pressure':ti,ab OR 'Hypertensia':ti,ab OR 'Arterial hypertension':ti,ab OR 'Systemic hypertension':ti,ab) AND ('Morning':ti,ab OR 'Daytime':ti,ab OR 'Sunlight':ti,ab) AND ('Evening':ti,ab OR 'Midnight':ti,ab OR 'Darkness':ti,ab OR 'Nighttime':ti,ab) AND ('Exercis*':ti,ab OR 'Train*':ti,ab)	All fields	N = 276
Web of Science	TS=("Hypertension" OR "Hypertensive" OR "HTN" OR "Elevated blood pressure" OR "High blood pressure" OR "Hypertensia" OR "Arterial hypertension" OR "Systemic hypertension") AND TS=("Morning" OR "Daytime" OR "Sunlight") AND TS=("Evening" OR "Midnight" OR "Darkness" OR "Nighttime") AND TS=("Exercis*" OR "Train*")	All fields	N = 208

Eligibility Criteria

The included trials had to study adult patients with hypertension undergoing an exercise intervention, comparing exercise conducted at different times of the day (primarily morning and evening). The trials to be considered suitable had to contain information about at least one of these aspects of clinical observation: post-exercise BP, systolic BP (SBP), diastolic BP (DBP), mean arterial pressure (MAP), 24-hour BP, and nocturnal BP. The trials to be included were restricted to being RCTs. Studies were excluded if they were observational in nature, non-randomized, animal studies, abstracts without full texts, review articles, or case reports. The list of the excluded studies is shown in Table 2. 

**Table 2:** List of excluded studies during the full-text screening step.

Study ID	Title	Reason for exclusion
Brito et al. (2015)	Exercise performed at different times of the day has different effects on ambulatory blood pressure, heart rate and arterial stiffness	Different population
Ramirez-Jimenez et al. (2022)	Aerobic exercise training improves nocturnal blood pressure dipping in medicated hypertensive individuals	No control group
De Brito et al. (2017)	Heart rate recovery improvement is greater after evening than morning aerobic training in treated hypertensive men	Irrelevant outcomes
Brito et al. (2016)	Influence of time of day on post-exercise hypotension might be different in hypertensives receiving different anti-hypertensive drugs: an exploratory study	Abstract
Boettcher et al. (2024)	Correction to: Exercise systolic blood pressures are unaffected by time of day in healthy young adults	Different population
Di Blasio et al. (2010)	Relationship between time of day and variation in blood pressure response to aerobic exercise	Different population
Kroenig et al. 1976	Blood pressure response to physical activity in hypertensive subjects at different times of the day	No control group
Ciolac et al. (2008)	Acute aerobic exercise reduces 24-hour ambulatory blood pressure levels in long-term-treated hypertensive patients	Different study design
Jimenez et al. (1994)	Hemodynamic and hemostatic responses to morning and evening exertion in systemic hypertension and implications for triggering of acute cardiovascular disease	Irrelevant outcomes
Pagonas et al. (2014)	The impact of aerobic exercise on blood pressure variability	Irrelevant outcomes
Fagard et al. (1995)	The role of exercise in blood pressure control: supportive evidence	No control group
Tanindi et al. (2015)	Blood pressure morning surge, exercise blood pressure response and autonomic nervous system	Different population
Caminiti et al. (2021)	Effects of 12 weeks of aerobic versus combined aerobic plus resistance exercise training on short-term blood pressure variability in patients with hypertension	Different study design
Rayamajhi et al. (2024)	Exercise as medicine; chronotherapy in hypertension management	Different study design
Li et al. (2024)	Effects of the timing of intense physical activity on hypertension risk in a general population: a UK-Biobank study	Different population
Alves et al. (2019)	Aerobic training decreases 24-hour and daytime ambulatory blood pressure in patients with resistant hypertension	Different study design
Lopes et al. (2021)	Aerobic exercise training reduces blood pressure, angiotensin II and oxidative stress of patients with resistant hypertension: the EnRiCH trial	Different study design
Ciolac et al. (2009)	Acute effects of continuous and interval aerobic exercise on 24-hour ambulatory blood pressure in long-term treated hypertensive patients	Different study design

Study Selection Process and Data Extraction

All identified records were exported into Rayyan (Rayyan Systems Inc., Cambridge, MA, USA), and duplicate citations were removed. The screening process comprised two stages: title and abstract screening and full-text screening of eligible studies. A two-reviewer process was used to screen the articles, and any differences in opinions were resolved through discussion. Data extraction was independently conducted by two reviewers using a data extraction form. Extracted data included characteristics of studies and patients, along with our outcomes of interest. In case of disagreement, it was resolved via consensus.

Risk-of-Bias Assessment

To assess the quality of the citations that were included, we applied the Cochrane Risk of Bias 2 (RoB 2) checklist. Two co-authors performed the assessment of the risk of bias, and disagreement on the ratings was resolved by discussion with the principal investigator. Authors assigned a risk level to each scale domain and to the overall quality of the selected publications from “low risk of bias,” over “some concerns risk of bias,” to “high risk of bias”. Disagreements were resolved through discussions.

Data Synthesis

Due to heterogeneity in study design, intervention protocols, and outcome reporting, a quantitative meta-analysis was not feasible. Therefore, findings were synthesized qualitatively using a structured narrative approach, grouping studies based on intervention duration (short-term vs long-term) and key clinical characteristics. For crossover trials, results from each intervention phase were interpreted within-study, accounting for the crossover design as reported by the original authors.

Results

Study Selection

**Figure 1:**
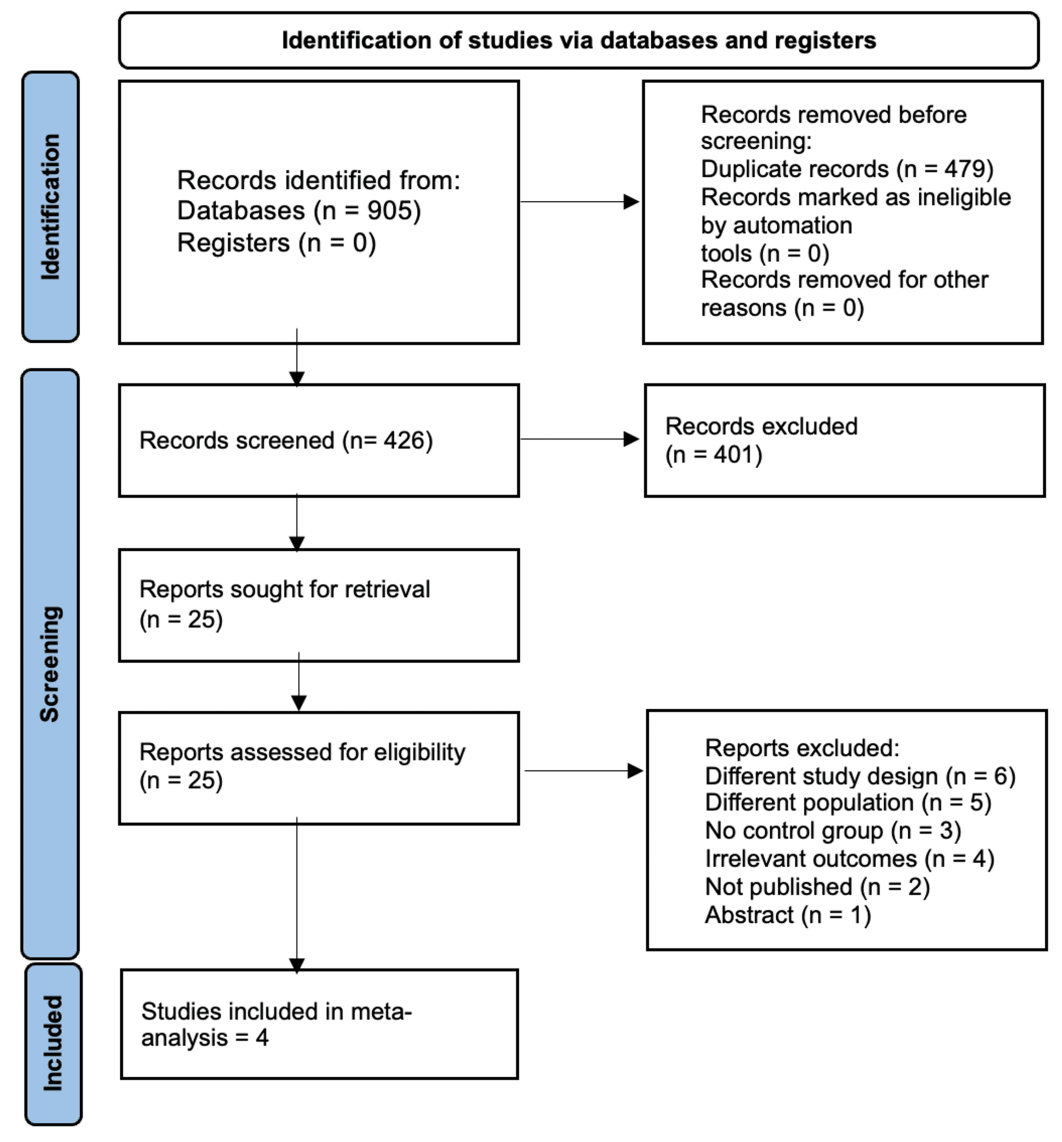
PRISMA flow diagram. PRISMA: Preferred Reporting Items for Systematic Reviews and Meta-Analyses.

Our search strategy identified 905 records from four databases. Deduplication identified 479 duplicates, resulting in 426 records eligible for screening. Screening identified 25 articles for full-text evaluation. Four articles out of 25 records met our study criteria, resulting in four studies with five reports meeting our study criteria. The PRISMA flow diagram is shown in Figure 1.

Study Characteristics

Four studies [11-14] were included in our study, comprising five reports [11-15]. Two trials tested long-term interventions conducted over 10 weeks. Both trials were parallel RCTs. Two trials tested the effect of short-term interventions on BP. All three trials were crossover RCTs. One study was a secondary analysis of a short-term outcome from a long-term trial [15]. Included trials and their characteristics are shown in Table 3.

**Table 3:** Characteristics of the included trials.

Study	Follow-up	Design	Sample size	Target population	Country	Exercise type
Brito et al. (2019) [11]	Long term	Parallel RCT	15 morning group; 15 evening group; 20 control	Hypertensive men age 30-65	Brazil	Intervention: aerobic exercise 3x weekly. Control: stretches 3x weekly
Brito et al. (2024) [13]	Long term	Parallel RCT	12 morning group; 11 evening group	Elderly (age ≥ 60) hypertensive patients	Brazil	Aerobic exercise 3x weekly
Park et al. (2005) [14]	Short term	Crossover RCT	9 patients with dipper HTN; 5 patients with non-dipper HTN	Hypertensive middle-aged adults	United States	Intervention: 30-minute treadmill walk. Control: rest
Azevêdo et al. (2017) [12]	Short term	Crossover RCT	11	Hypertensive middle-aged women	Brazil	Intervention: exercise performed in the morning, night, or fractionated sessions. Control: rest

Risk-of-Bias Assessment

**Figure 2:**
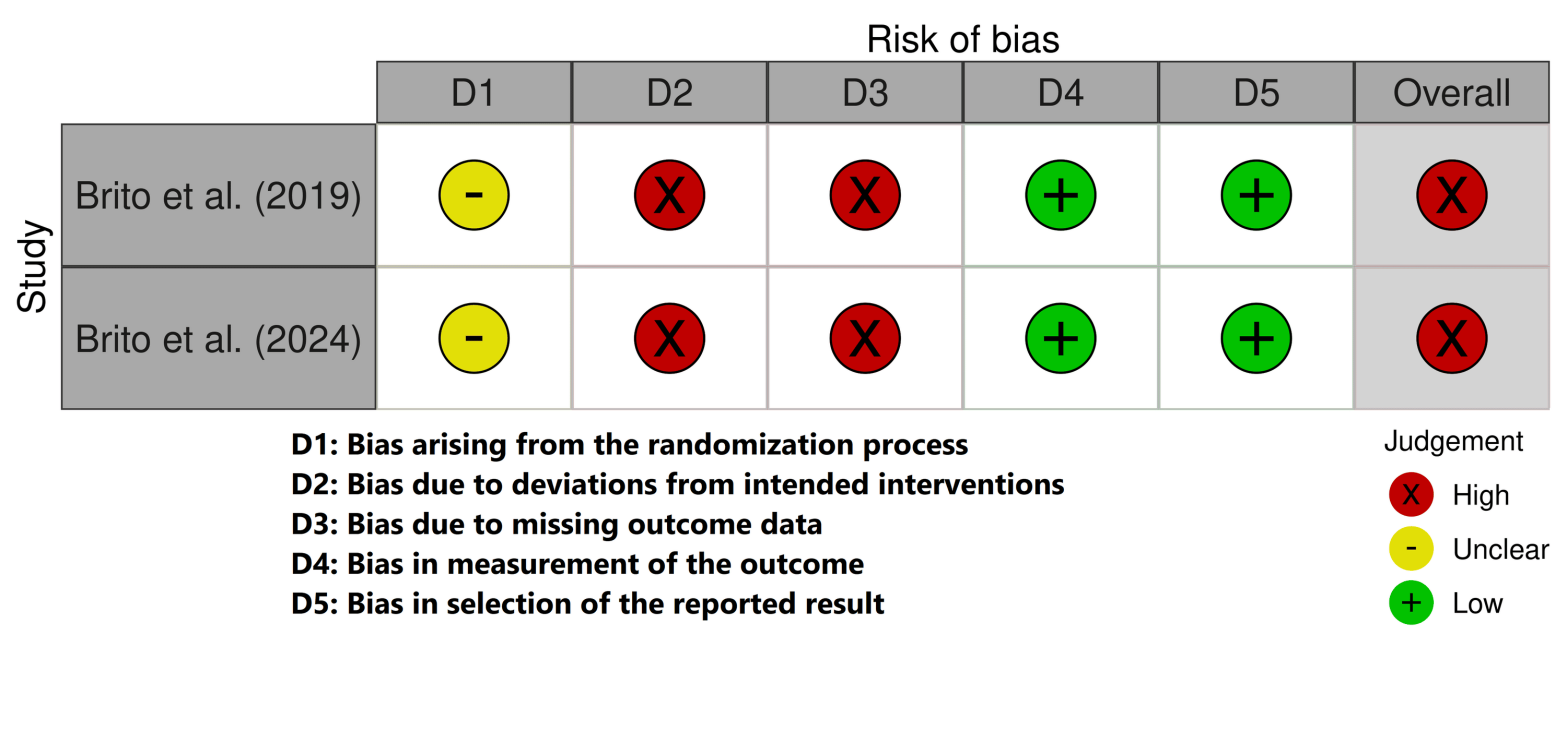
Risk of bias for long-term interventions. Source: [11,13].

**Figure 3:**
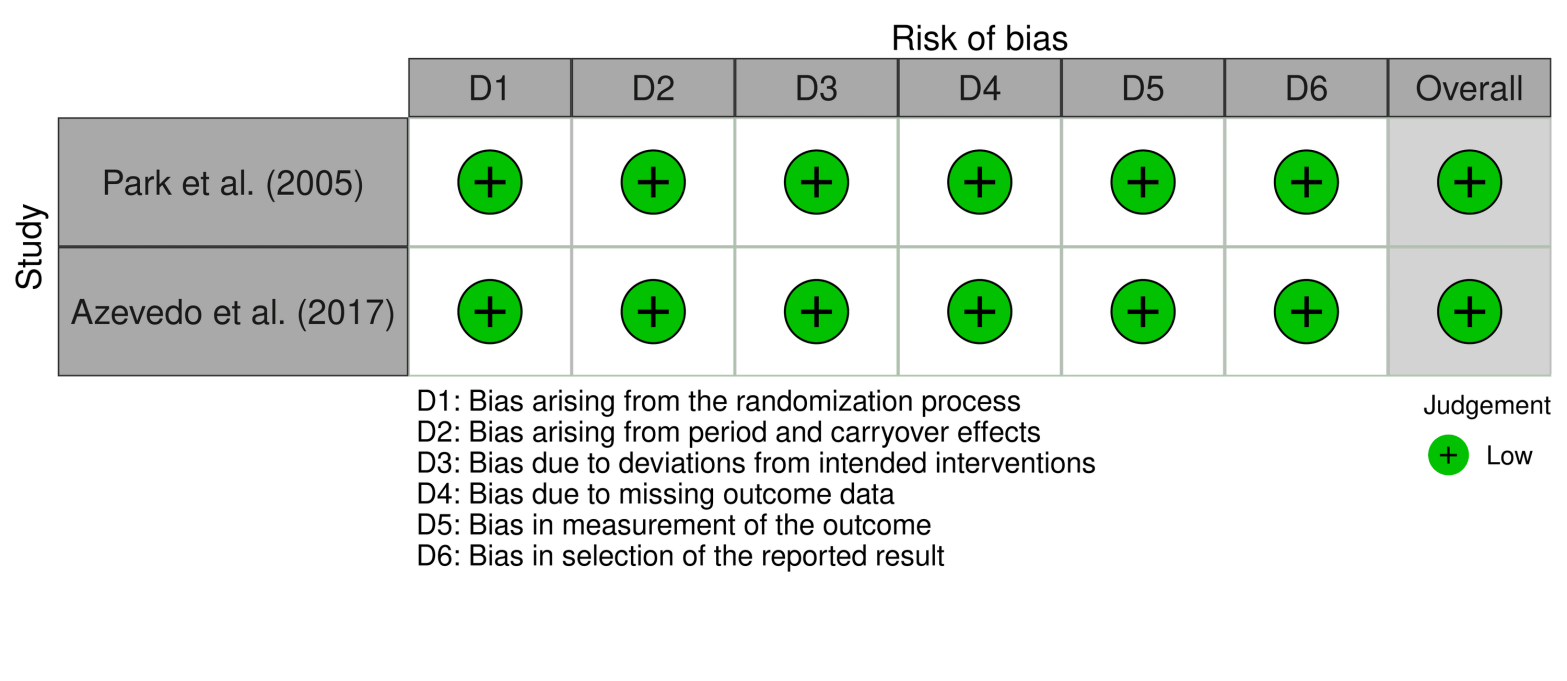
Risk of bias for short-term interventions. Source: [12,14].

All five trials underwent an assessment of bias. Parallel RCTs were assessed by the revised RoB 2 tool, while crossover RCTs were evaluated using a modified version of RoB 2 for crossover designs. Both long-term intervention studies were judged to have a high risk of bias (Figure 2), whereas the two crossover trials were assessed as having a low risk of bias (Figure 3).

Effects of Morning and Evening Exercises on BP in Long-Term Interventions

Two studies compared the effect of morning and evening exercises in long-term interventions across multiple weeks. An RCT done by Brito et al. was conducted over a 10-week period on hypertensive men [11]. Participants (n = 50) were divided into three groups randomly: a morning exercise group (n = 15, exercise done between 7:00 and 9:00 A.M.), an evening exercise group (n = 15, exercise done between 6:00 and 8:00 P.M.), and a control group (n = 20). The control group was further divided into morning and exercise control groups, where each group would do stretching exercises in each period, respectively.

Morning evaluations of SBP and DBP showed that the morning therapy group did not significantly decrease SBP compared to the control group. On the other hand, evening therapy significantly decreased SBP (-5 ± 6 mmHg) in comparison to both the control group and the morning therapy group. No significant changes in morning DBP were found between the groups. Evening evaluations of SBP and DBP showed similar trends to the morning evaluation. Evening therapy again significantly decreased SBP in comparison to both the control group and the morning therapy group, while no differences in DBP were observed. Evening therapy also significantly decreased 24-hour DBP, asleep DBP, and mean BP compared to the other groups. No differences in 24-hour SBP, asleep SBP, awake SBP, and awake DBP were noticed between the groups. Table 4 summarizes the results from this trial. 

**Table 4:** Summary of changes in multiple blood pressure (BP) readings. **Significant difference compared with both the control and morning therapy groups. Source: [11].

Variable	Assessment time	Control (C)	Morning training (MT)	Evening training (ET)
Clinic SBP	Morning evaluation	Not reported	Not reported	-5 ± 6 mmHg **
	Evening evaluation	Not reported	Not reported	-8 ± 7 mmHg **
Clinic DBP	Morning evaluation	Not reported	Not reported	Not reported
	Evening evaluation	Not reported	Not reported	Not reported
24-hour BP	24-hour SBP	Not reported	Not reported	Not reported
	24-hour DBP	Not reported	Not reported	-3 ± 5 mmHg **
Asleep BP	Asleep SBP	Not reported	Not reported	Not reported
	Asleep DBP	Not reported	Not reported	-3 ± 4 mmHg **
Awake BP	Awake SBP	Not reported	Not reported	Not reported
	Awake DBP	Not reported	Not reported	Not reported
Resting Mean BP	Morning evaluation	Not reported	Not reported	-4 mmHg **
	Evening evaluation	Not reported	Not reported	-7 mmHg **

Another RCT by Brito et al. compared morning and evening exercises [13]. However, the trial was focused on elderly patients (both male and female) with hypertension. A total of 23 patients completed the intervention, and their data were reported in the final publication. Participants were randomized into morning (n = 12) or evening (n = 11) groups. Morning sessions were from 7:00 to 10:00 A.M. Evening sessions were from 5:00 to 8:00 P.M. Patients completed a 10-week intervention of aerobic training.

The trial showed that only evening therapy significantly decreased mean BP (evening vs morning, -9 ± 11 vs -1 ± 8 mmHg, respectively) and SBP (evening vs morning, -13 ± 10 vs. -1 ± 11 mmHg, respectively) compared to the baseline and morning therapy group. Both evening and morning sessions significantly decreased DBP compared to the initial baseline, with a decrease of -4 ± 11 and -2 ± 10 mmHg, respectively. No session was superior to the others in terms of decreasing DBP. Heart rate did not decrease significantly in either the evening or morning groups. Table 5 summarizes the BP changes for this trial. 

**Table 5:** Blood pressure (BP) changes. *Significant change compared to initial baseline. **Significant change compared to the morning therapy group. Source: [13].

Variable	Morning training (MT)	Evening training (ET)
Systolic BP	-1 ± 11 mmHg	-13 ± 10 mmHg **
Diastolic BP	-2 ± 10 mmHg*	-4 ± 11 mmHg*
Mean BP	-1 ± 8 mmHg	-9 ± 11 mmHg **

Effects of Morning and Evening Exercises on BP and Sympathetic Markers in Short-Term Interventions

A crossover RCT by Park et al. was conducted on 14 hypertensive adults and compared morning (6:00-8:00 A.M.) and evening (5:00-7:00 P.M.) exercises [14]. However, it compared between patients who had dipper hypertension (n = 9), defined as ≥10% decrease in average nighttime SBP, and patients who had non-dipper hypertension (n = 5). Using the crossover trial methodology, all patients went through four groups: morning exercise, morning rest, evening exercise, and evening rest. The respective control and exercise groups were up to four days apart for each subject. The two exercise groups were done at least a week apart to remove any training effect on BP. Their results show that evening exercise caused a larger decrease in nighttime SBP in non-dippers (-11.5 ± 3.85 mmHg) compared to dippers (-0.3 ± 1.7 mmHg). Morning exercise produced similar daytime systolic BP reductions in dippers (-5.6 ± 2.59 mmHg) and non-dippers (-6.0 ± 1.23 mmHg). Both morning and evening exercise exhibited similar reductions in 24-hour BP in dippers (morning -5.56 ± 2.27 mmHg, evening -0.11 ± 2.29 mmHg) and non-dippers (morning -7.22 ± 2.10 mmHg, evening -7.0 ± 3.16 mmHg).

Using data from a previously mentioned clinical trial (n = 50) [11], a post hoc analysis was done to compare PEH between morning and exercise groups in patients on antihypertensive therapy. Data of patients on angiotensin-converting enzyme inhibitors (ACEi; n = 14) and angiotensin II receptor blockers (ARBs; n = 15) from the previous trial were further analyzed. In the ACEi group, BP decreased significantly after morning exercise (102 ± 10 vs 99 ± 10 mmHg). In the ARBs group, BP decreased after evening exercise (103 ± 7 vs 101 ± 6 mmHg). In terms of PEH, mean BP in the morning and evening groups were similar in patients on ACEi (evening vs morning, -2 ± 5 vs -3 ± 4 mmHg, respectively). Patients on ARBs noticed a significantly larger decrease in the evening group compared to the morning group (evening vs morning, -4 ± 5 vs. -1 ± 5, respectively).

Azevêdo et al. conducted a crossover RCT on 11 hypertensive middle-aged women. This trial not only compared morning and evening exercises but also included a “fractionated exercise” group. This group conducted 50% of the intended workout in the morning session, and the rest of the workout in the evening session. The main outcomes were PEH and BP reactivity after exercise. All 11 participants went through four groups (morning, evening, control, and fractionated). SBP and DBP were measured 15, 30, 45, and 60 minutes after each exercise session. BP reactivity was measured by a cold reactivity test at 60 minutes. Their results showed that the SBP decrease in the morning session was significantly higher at 15, 45, and 60 minutes post-exercise (-7.1 ± 12.1, -10.7 ± 12.9, and -6.8 ± 11.5 mmHg, respectively) compared to the control group. Night sessions also caused a significantly larger decrease in SBP at 15 and 45 minutes post-exercise (-6.6 ± 7.7 and -6.3 ± 5.1 mmHg) compared to the control group. Fractionated exercise was not shown to be superior in decreasing SBP compared to the control group. No significant differences in DBP were noted between the groups. Only morning sessions significantly attenuated BP reactivity for both SBP (~44%) and DBP (~59%) relative to the control group. Table 6 summarizes the overall results. 

**Table 6:** Summary of results. BP: blood pressure, SBO: systolic blood pressure, DBP: diastolic blood pressure, ARBs: angiotensin II receptor blockers, ACEi: angiotensin-converting enzyme inhibitors.

Study	Subgroups	Preferred time to exercise	Notes
Long-term interventions			
Brito et al. (2019) [11]	-	Evening	Evening exercise was superior in decreasing mean BP, SBP, asleep BP, and 24-hour DBP
Brito et al. (2024) [13]	-	Evening	Evening exercise was superior in decreasing mean BP, SBP, and DBP
Short-term interventions			
Park et al. (2005) [14]	Dipper hypertensives	Neutral	Similar decreases in 24-hour BP in both sessions
	Non-dipper hypertensives	Evening	Superior decrease in nighttime BP
Brito et al. (2019) [11]	Patients on ARBs	Evening	Significantly larger post-exercise hypotension
	Patients on ACEi	Neutral	Similar post-exercise hypotension regardless of time
Azevêdo et al. (2017) [12]	-	Morning	Larger decrease in SBP and attenuation in BP reactivity

Discussion

Summary and Findings

Our systematic review shows that evening exercise is more likely to be effective on a long-term basis in reducing SBP and MAP in male hypertensive populations, with the reduction in nocturnal BP being particularly significant. Short-term interventions showed conflicting results, with multiple trials showing either morning or evening exercise superiority. The only trial that was limited to females suggested that morning exercise causes a larger decrease in SBP.

Our systematic review found that the long-term interventions of evening exercise are more effective for reducing clinic and ambulatory SBP in hypertensive men and elderly patients compared to morning exercise. These findings are supported by the work of Arciero et al., who conducted a 12-week multimodal training trial and found that evening exercise was more effective for lowering SBP in healthy men [16]. This suggests that, in hypertensive men, performing exercise in the evening may provide a more favorable physiological environment for vascular remodeling to occur. This might be related to the body’s natural circadian rhythm, in which sympathetic nervous system activity and vascular tone gradually decline as the day progresses, leading to less vasoconstriction in the evening hours. This can explain the prolonged decrease in SVR when exercise is performed during this period of reduced sympathetic drive. This might give the cardiovascular system a more favorable window for endothelial recovery, improved arterial compliance, and ultimately more effective long-term adaptations in blood pressure regulation [17]. Future studies with longer follow-up periods and assessments of physiological mechanisms are necessary in order to provide higher quality evidence on the effect of evening exercise on BP.

On the other hand, trials assessing the effect of short-term exercise interventions showed conflicting results. Our findings also align with the recent meta-analysis by Sevilla-Lorente et al., who analyzed 11 studies and concluded there was no significant difference between morning and evening acute exercise for SBP [18]. A potential reason for the wide variation in results is due to the difference in exercise regimens implemented in each trial. It is known that exercise intensity can affect PEH. In addition, other factors, including gender, body mass index, and age, can affect PEH. This might suggest that the effect of time of exercise manifests properly in long-term interventions [19,20].

A trial in our study suggested that evening exercise can restore nocturnal dipping in non-dipper hypertensive patients. This is a significant finding given that non-dipping is an independent predictor of cardiovascular mortality. These results are consistent with the chronobiological hypothesis, where evening exercise extends vasodilation into the sleep period, effectively substituting for the absent natural dip [17]. Conversely, these findings appear to contradict Fairbrother et al., who reported that early morning exercise (7:00 AM) invoked a greater dip in nocturnal SBP compared to afternoon or evening sessions [6]. This could be due to the health status of the participants. Fairbrother et al. studied pre-hypertensive subjects who presumably retained some degree of normal autonomic variability. In contrast, the participants in our study were established non-dipper hypertensives, a group characterized by sustained nocturnal sympathetic overdrive. It is suggested that morning exercise improves sleep quality by increasing deep sleep, which could be particularly beneficial for people with slightly elevated blood pressure or pre-hypertensives, whereas evening exercise is more effective for people with non-dipping hypertension because it can help to mechanically break the sympathetic dominance. Thus, dipping status should be assessed before prescription: morning exercise may prevent the loss of dipping in pre-hypertensives, while evening exercise is required to restore it in non-dippers [6,21].

While our findings in men favored evening exercise, hypertensive women appeared to benefit more from morning exercise, particularly in terms of reducing BP reactivity to stress. This finding is supported by Arciero et al., who found that, in women, morning exercise significantly reduced abdominal fat and BP, whereas evening exercise resulted in greater muscular performance gains but less robust BP reductions [16]. This sex-divergence suggests that the mechanisms of BP reduction may differ between men and women. In women, morning exercise may function by optimizing cardiac autonomic balance and reducing central sympathetic outflow throughout the day, buffering against stress-induced spikes. In men, the primary benefit of evening exercise appears to be peripheral, mediated by reductions in vascular resistance [22]. However, while there are sex differences in autonomic and vascular responses to exercise, no study has conclusively connected these differences to better outcomes when exercise is scheduled in the morning versus evening, depending on sex. Consequently, the "one-size-fits-all" approach to exercising timing is insufficient. We recommend further studies to determine if morning exercise should be prioritized for hypertensive women, particularly those with high stress reactivity, while evening protocols are maintained for men to target basal vascular resistance.

Our analysis showed that patients taking ARBs experienced a more pronounced hypotensive response to evening exercise, whereas those on ACEi did not exhibit the same degree of benefit. This is an important concern considering the prevalence of these drugs. This finding could be explained when considering the pharmacodynamics of the renin-angiotensin system (RAS). ACEi increases bradykinin levels, which promote vasodilation via nitric oxide and prostacyclin pathways [23]. Conversely, ARBs, although they do not increase bradykinin, have been shown in a meta-analysis of randomized trials to improve peripheral endothelial function, which suggests that ARBs can maintain vascular responsiveness and structural function over time [24]. This interaction suggests that exercise guidelines should be adjusted based on the patient's pharmacological profile. Future studies are recommended to determine how ACEi and ARBs modulate morning versus evening exercise responses, incorporating mechanistic vascular biomarkers, dipping-status phenotyping, and sex-specific variations to identify individualized exercise prescriptions for hypertensive patients.

The primary limitation of this systematic review is the small sample size. Most included studies had sample sizes of fewer than 50 participants, reducing statistical power and the ability to generalize findings. Additionally, there was significant heterogeneity in exercise protocols (cycle ergometer vs treadmill, continuous vs fractionated) and intensity, which complicates direct comparisons. Finally, the majority of long-term data comes from a single research group, which has a high risk of bias, highlighting the need for replication by independent centers to confirm the generalizability of the study findings.

## Conclusions

This systematic review found that the timing of exercise could play a role in controlling BP in hypertensive patients. Evening exercise showed greater long-term reductions in BP, while short-term interventions yielded inconclusive results. These findings highlight an important area for future studies. In addition, our findings highlight the potential value of personalizing the time of exercise for the optimal benefit in BP reduction.
